# Genetic Polymorphism in Extracellular Regulators of Wnt Signaling Pathway

**DOI:** 10.1155/2015/847529

**Published:** 2015-04-05

**Authors:** Garima Sharma, Ashish Ranjan Sharma, Eun-Min Seo, Ju-Suk Nam

**Affiliations:** ^1^Institute for Skeletal Aging & Orthopaedic Surgery, Hallym University-Chuncheon Sacred Heart Hospital, Chuncheon-si, Gangwon-do 200-704, Republic of Korea; ^2^Amity Institute of Nanotechnology, Amity University Uttar Pradesh, Noida, Uttar Pradesh 201313, India; ^3^Adbiotech Co., Ltd., Chuncheon-si, Gangwon-do 200-880, Republic of Korea

## Abstract

The Wnt signaling pathway is mediated by a family of secreted glycoproteins through canonical and noncanonical mechanism. The signaling pathways are regulated by various modulators, which are classified into two classes on the basis of their interaction with either Wnt or its receptors. Secreted frizzled-related proteins (sFRPs) are the member of class that binds to Wnt protein and antagonizes Wnt signaling pathway. The other class consists of Dickkopf (DKK) proteins family that binds to Wnt receptor complex. The present review discusses the disease related association of various polymorphisms in Wnt signaling modulators. Furthermore, this review also highlights that some of the sFRPs and DKKs are unable to act as an antagonist for Wnt signaling pathway and thus their function needs to be explored more extensively.

## 1. Introduction

**Figure 1:**
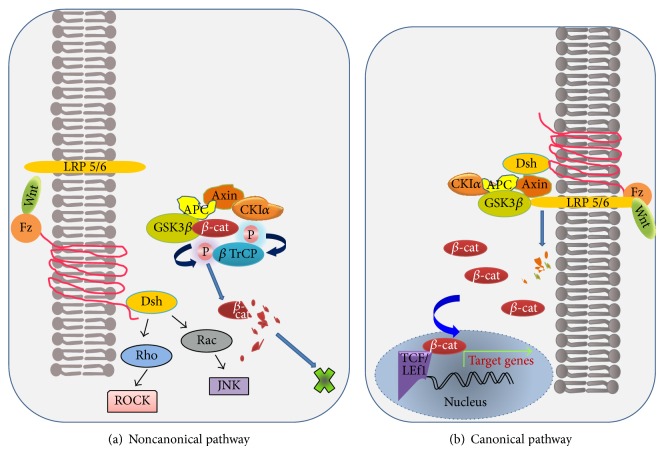
Canonical and noncanonical Wnt signaling pathways.

The Wnt family, a group of secreted glycoproteins, directs cell proliferation and polarity as well as determining the fate of a cell during embryonic development through a series of signaling transduction pathways [1]. The members of human Wnt family consist of 19 evolutionarily conserved glycoproteins having 22 or 24 cysteine residues [2]. The mechanisms followed by Wnt signaling molecules are either through canonical pathway (cell fate determination) or via noncanonical pathway (control of cell movement and tissue polarity) (Figure 1). Signal transduction during canonical Wnt pathway is mediated through the family of frizzled (FZD) receptor and low density lipoprotein related protein 5 (LRP5)/6 LRP6 coreceptors, activating *β*-catenin signaling cascade [3, 4]. The Wnt signaling pathway continues after Wnt ligand binds and forms a complex with FZD and LRP6/5 together with phosphorylated scaffolding protein dishevelled (DVL). Thereafter, phosphorylation of LRP6 leads to binding of AXIN complex to the receptors, thus disabling AXIN complex to degrade *β*-catenin. On the contrary, the absence of Wnt ligand leads to constant degradation of cytoplasmic *β*-catenin protein via AXIN complex [5]. Scaffold protein AXIN and the tumor suppressor gene product adenomatous polyposis coli (APC) form AXIN complex together with casein kinase 1 (CK1) and glycogen synthase kinase 3 (GSK3). CK1 and GSK3 phosphorylate *β*-catenin at amino terminus followed by *β*-catenin recognition and ubiquitination by *β*-transducing repeats-containing proteins (*β*-TrCp), an E3 ubiquitin ligase subunit directing proteasome mediated degradation [6]. As a result, *β*-catenin is unable to reach the nucleus and subsequently Wnt targeted genes are repressed by DNA-bound T-cell factor/lymphoid enhancer factor (TCF/LEF) family of proteins. In noncanonical signaling pathway, Wnt ligand binds to the receptors of FZD family and receptor tyrosine kinase-like orphan receptor 2/receptor tyrosine kinase (ROR2/RYK) coreceptors to form a complex. Upon stimulation by the receptors, DVL is activated leading to sequential activation of the Rho family of GTPases (small G proteins such as RhoA, RhoU, RAC, and CDC42) and JNKs. Another noncanonical Wnt signaling pathway involves activation of Wnt/Ca^++^ signaling cascade.

**Figure 2:**
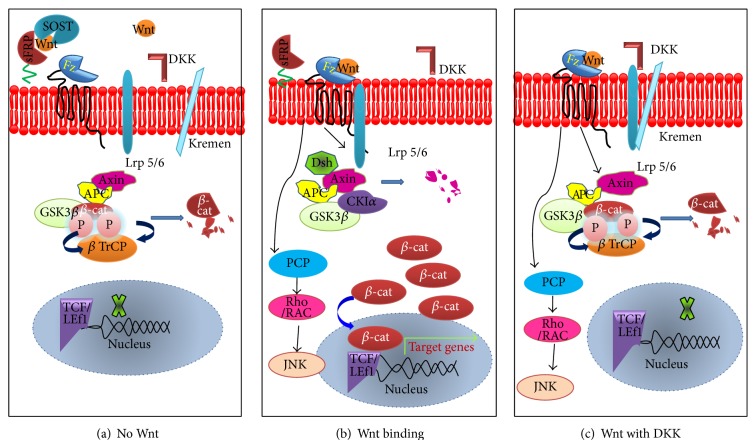
Role of antagonists in Wnt signaling pathway.

Various natural inhibitors of Wnt signaling pathway have been identified till date, which can antagonize or regulate Wnt signaling pathway (Figure 2). The mechanism of Wnt antagonists has been highlighted by various developmental studies performed in various chick and Xenopus models [7, 8]. Broadly, antagonists of Wnt signaling pathway are divided into two classes depending on their functional mechanism, that is, secreted frizzled-related proteins (sFRPs) and Dickkopfs (DKKs). The members of sFRP class can bind to Wnts and thus regulate the association of Wnt ligands to their transmembrane receptors, inhibiting both canonical and noncanonical signaling pathways. The sFRP class constitutes sFRP family proteins: WIF-1 and Cerberus. Another class of antagonists, that is, DKK class, binds to LRP5/LRP6 component of the Wnt receptor complex to inhibit canonical Wnt signaling and constitutes DKK family proteins and sclerostin [9]. Besides antagonists, Wnt signaling pathway is also activated and regulated by some secreted proteins acting as agonists, for example, R-spondin (Rspo) [10].

As mentioned earlier Wnt signaling is one of the most critical pathways for cellular development, even in adult tissues; henceforth, the polymorphism in genes of Wnt signaling modulators can be a foremost cause for various types of associated diseases. The present review highlights disease associated polymorphism studies in some of the Wnt signaling modulators. Summarizing the role of polymorphism in Wnt signaling modulators can open a new spectrum to understand the origin of various related diseases and to trace out novel pathways for potential therapeutics.

## 2. Polymorphism in* SOST*/Sclerostin

Sclerostin is a* SOST* gene encoded secreted glycoprotein containing cysteine knot which acts as a Wnt antagonist and regulates skeletal mineralization [11, 12]. Recently, expression of SOST mRNA has also been reported in various cells, including liver, blood vessels, and kidneys [13], though these organs are devoid of sclerostin protein. The major expression of sclerostin protein has been found in skeletal tissues such as articular chondrocytes [14] and cementocytes [15]. Even during inflammatory diseases like periprosthetic osteolysis, it has been shown to be induced in osteoblasts by cytokines like tumor necrosis factor alpha (TNF*α*) [16]. In osteocytes and osteoblasts, sclerostin functions as a Wnt/*β*-catenin signaling antagonist by binding to LRP5/6 receptors and thus inhibiting bone formation [17].* SOST* genes have been associated with osteoporotic fractures and bone mineral density (BMD) [18–20]. However, in a different study, performed on 619 perimenopausal Scottish women, single nucleotide polymorphism (SNP) in* SOST* gene region was not found associated with high or low BMD at lumbar spine [21]. Similarly, in a genotypic study of 652 Slovenian populations BMD was not associated with -1397/-1396insGGA* SOST *polymorphism [22]. On the contrary, a genome wide linkage analysis of Chinese cohort revealed that the genetic polymorphism in sclerostin domain-containing protein 1 (SOSTDC1) has correlation with bone mass density [23]. Although difference between* SOST *genotype in correspondence to the allele dose effect was also observed concluding the necessity for large sample size, Li et al., 2008, showed increases in BMD in* SOST *knockout mice [24]. It has been earlier reported that loss of function mutations in the* SOST* gene leads to monogenic bone disorder, sclerosteosis (Online Mendelian Inheritance in Man ID: 269500), characterized by hyperostosis all over the skeleton (massive bone overgrowth) [25, 26]. Additionally, another mutation (52 kb deletion) at 35 kb downstream of the* SOST *gene causes van Buchem disease with similar disease phenotype [27, 28]. Uitterlinden et al., 2004 [29], hypothesized that polymorphism in* SOST* gene region influences the function of sclerostin causing variations in BMD in a subset of the population. Further, to verify their hypothesis they observed eight polymorphisms within the region of the* SOST *gene, which were in relation to BMD observed in 1,939 elderly Dutch white. According to their results, a decrease in BMD of spine/femoral neck in Dutch white females was associated with a 3 bp insertion in the* SOST *promoter region (*SOST* gene region polymorphism 3) and increase in BMD of spine/femoral neck in Dutch white males was associated with G variant of* SOST*-region polymorphism 9, that is, van Buchem deletion region. Recently, two BMD associated SNPs (rs851054 and rs851056) were also identified in 5′ region of* SOST *gene [18]. Styrkarsdottir et al., 2009, also identified three SNPs that were in association with BMD and were located at 3′* SOST* gene region between 23 kb and 57 kb (rs1513670, rs7220711, and rs1107748) [30]. Evidence of association between BMD and −9247 polymorphism (rs1230399) at 5′* SOST *gene region was also proved by a gene wide tag SNP association study performed on Chinese population (1243 subjects) with low and high BMD [31].

## 3. Polymorphism in Secreted Frizzled Related Protein (sFRP)

Based on sequence homology, the five members of sFRP family (sFRP1 to sFRP5) are divided into two subgroups. sFRP1, sFRP2, and sFRP5 constitute one subgroup while sFRP3 and sFRP4 are in another subgroup. These proteins are soluble cysteine rich and bind directly to Wnt molecules antagonizing Wnt signaling pathway [38]. sFRP3, also known as FrzB, was the first purified chondrogenic factor from cartilage [39]. Conflicting roles of sFRPs have recently been reported, such as sFRP1 that acts as an agonist,* in vitro,* at low concentration [40] while sFRP1 promotes angiogenesis in a chick chorioallantoic membrane model [41]. The role of* sFRP3* in the etiology of osteoarthritis has been discussed in many reports. Replication studies among Caucasian women showed two SNPs in* sFRP3/FrzB* gene, R200W (rs288326) and R324G (rs7775), may elevate knee osteoarthritis [32]. In another SNP analysis, association of these haplotypes with hip osteoarthritis have also been shown in a large population of postmenopausal Caucasian women [33]. In addition, the authors considered the contribution of SNPs and suggested them as a biomarker for osteoarthritis. Transfection studies in human embryonic kidney 293 cells (HEK293), performed by Loughlin et al., 2004, [42] displayed that substitution of highly conserved positively charged arginine residues reduces the antagonizing ability of sFRP3/FrzB. sFRP3, as expressed by chondrocytes, mediated inhibition of Wnt signaling pathway is known to be vital for maintaining the integrity of cartilage-bone junction. Therefore, R324G SNP in* sFRP3* results in structurally occult hip dysplasia leading to osteoarthritis. Further, they revealed that the G allele of R324G variant is in close association with hip replacement in elderly females. Furthermore, Min et al., 2005, also showed increased incidence of G allele of this variant in individuals with hip radiographic osteoarthritis. According to them individuals as G allele carrier have a high risk for hip radiographic osteoarthritis [43]. Similar results were also observed in a study performed in Spanish cohort observing association between FRZB and hip, knee, and hand osteoarthritis. Group observed increased frequency of R324G in patients with multiple joint osteoarthritis and in females with hip osteoarthritis [34]. Besides osteoarthritis, rs7775 SNP in* sFRP* gene has been shown to exhibit a strong association with breast cancer. SNP rs7775 was found in exon 6 and was observed to encode arginine (CGC) or glycine (GGC) in Saudi women [35].

## 4. Polymorphism in Dickkopf

Another class of Wnt antagonist is cysteine rich secreted protein known as Dickkopf (DKK-1 to -4) which binds to LRP5/6 receptor and Kremen 1/2 (transmembrane protein) and inhibits Wnt signaling pathway. In human the gene for DKK-1 is located in 10q11.2 position of chromosome (NM_012242.2). As a result, the canonical pathway of Wnt signaling is inhibited [44, 45]. Studies in human DKK-1 showed a multifaceted effect on proliferation and differentiation of various cells like human adult bone marrow cells [46], adipocytes [47], and osteoblasts [48]. Moreover, the elevation of the DKK-1 level by glucocorticoids in osteoblasts that may lead to osteoporosis has also been observed [49]. In addition, Tian et al., 2003, observed the association of lytic bone lesions and elevated DKK-1 level in patients with multiple myeloma [50]. Reports suggest that the induction of DKK-1 is dependent on p53 and thus DKK1 acting as Wnt antagonist may lead to p53 tumor suppression [51]. Shou et al., 2002, also suggested the proapoptotic role of DKK-1 which links oncogenic Wnt and p53 tumor suppressor pathways [52]. Various reports showed antitumor effect of DKK-1 on different cell lines like HeLa cervical cancer cells [53, 54], colon cancer cells [55, 56], breast cancer cells [57, 58], renal cancer cells [59], and so forth.

Ralston et al. [60] suggested the association of* DKK-1* gene containing chromosomal region 10q21 with BMD in men through genome wide linkage scan. The influence of genetic variation in DKK-1 on hip geometry, BMD, and bone turnover has been studied earlier by Piters et al., 2010 [36]. They selected three SNPs (rs2241529 (G/A), rs1569198 (A/G), and rs1991392 (A/C)) using HapMap within 13.53 kb DKK-1 region and studied a group of 783 Caucasian men of 20–29 years. According to them, rs1569198 had significant association with hip axis length (HAL) and was independent of BMD and height. Results observed were further confirmed by haplotype analysis indicating increased risk of hip fracture in the general population. However, they further concluded that all the variants do not influence BMD or bone turnover marker, in chosen subjects, which was later on supported by other reports [61]. For the first time, the association of SNP rs6485350 in* DKK-3* gene and rs3763511 in* DKK-4* with breast cancer risk was studied by Alanazi et al., 2013 [35]. Study reported 2-fold reduced breast cancer risk in women with GG genotype as compared to AA genotype in case of* DKK-3* gene SNP. Furthermore, GG genotype and AG genotype showed enhanced protection against estrogen receptor positive tumor and estrogen receptor negative tumor progression, respectively. Conversely, SNP in* DKK-4* gene was strongly associated with age independent increased breast cancer risk of estrogen receptor negative tumor. In order to determine the relationship between polymorphism in* DKK* genes and renal cancer Hirata et al., 2009 [37], examined 210 renal cancer positive patients (145 male and 65 female). Through PCR-RFLP and direct sequencing genotype of SNP rs17037102 and rs419559, rs447372 in* DKK-2*, rs3206824, rs11022095, rs1472189, rs7396187, and rs2291599 in* DKK-3*, and rs2073664 in* DKK-4* were analyzed. Results demonstrated significant association of* DKK-3* rs1472189 C/T with renal cell carcinoma and SNP rs17037102 G* DKK-2* may contribute to increased survival rate in patients, after radical nephrectomy.

## 5. Wnt Inhibitory Factor 1

Another evolutionary conserved modulator protein of Wnt signaling is Wnt inhibitory factor 1 (Wif 1). It is similar to sFRPs and inhibits Wnt signaling by binding to Wnt proteins. Wif 1 contains epidermal growth factor like repeats (EGF repeats) and Wif domain which binds to Wnt proteins [62, 63]. As reported, Wif 1 is responsible for tumor suppression and epigenetic silencing and may lead to increased risk of cancer by modulating Wnt signaling pathway. Studies showed increased Wif 1 expression in C2C12, MC3T3-E1, and KS483 cells during BMP-2 induced osteoblasts differentiation suggesting the role of Wif 1 as modulator of osteoblasts differentiation and maturation [64]. Various studies have demonstrated that tumorigenesis gene silencing can be a result of abnormal methylation in promoter region of tumor suppressor genes [65, 66]. Kawakami et al., 2009, reported the downregulation of Wif 1 by promoter hypermethylation in renal cancer cells [67]. In addition, hypermethylation of* Wif 1* gene promoter region was found to downregulate Wif 1 gene in prostate cancer cell lines. Further, a decreased motility and invasiveness of prostate cancer cells were reported after restoration of Wif 1 expression [68]. Correlation of the reduced Wif 1 expression due to* Wif 1* promoter hypermethylation and increase in bladder tumor has also been reported by other research groups [69, 70].* Wif 1* gene has also been reported to be involved in osteosarcoma. Study performed by Kansara et al. revealed that in primary human osteosarcoma promoter hypermethylation leads to* Wif 1* gene silencing, which results in loss of differentiation and increased cell proliferation [71]. Taking into account the importance of* Wif* gene in regulation of tumorigenesis and any probability of existence of polymorphism in* Wif* gene, which may result in deleterious effect, makes it a probable candidate for requiring urgent studies for verifying its role in regulation of Wnt signaling pathway.

## 6. R-Spondins

R-spondins are a newly discovered, cysteine rich and containing a thrombospondin type 1 domain/repeat-1, secreted protein family consisting of 4 members (Rspo1-4). Recently, Rspo has been reported to act as modulators of Wnt signaling pathway. Rspo has been demonstrated to upregulate the Wnt signaling pathway by stabilizing cytosolic *β*-catenin [72, 73]. This is achieved by the binding of Rspo to LRP6 and thus disrupting LRP6-DKK complex, which further enhances the deactivation of canonical Wnt signaling [10, 74]. All the members of Rspo family have common structural organization and share around 60% sequence homology [75]. Recently, few studies reported that Rspo modulates Wnt signaling pathway through G protein-coupled receptors called leucine-rich repeat-containing G protein-coupled receptor (LGR) [76, 77]. Till date, three types of LGRs (LGR4-6) have been identified. As 7 transmembrane receptors, LGRs consist of leucine-rich repeats at extracellular N-terminal domain and are capable of binding to all four Rspo proteins [78]. Studies suggested that LRP/Wnt/Fz complex interacts with LRG-Rspo complex [79, 80]. Although the functional mechanism of Rspo mediated Wnt signaling has been discussed in some reports, yet, the exact mechanism of action for Rspo still remains to be elucidated. The association of Rspo family and bone metabolism has been documented in some studies, signified by the high level of Rspo protein expression in skeletal tissues at developmental stages and lacking of Rspo resulting in defected skeletal formation [81, 82]. Lately, mutations in* Rspo4* have been reported and were found associated with the absence of finger and toe nails (autosomal recessive anonychia/hyponychia) (OMIM 206800) in humans [83–86]. Khan et al., 2012, identified mutations (c.178C>T (p.R60W), c.353G>A (p.C118Y), and c.3G>A (p.M1I)) within chromosome 20p13 at Rspo4 locus after genotyping three anonychia/hyponychia Pakistani families using microsatellite markers. Study expanded* RSPO4* mutations related with anonychia/hyponychia to 17 and all of them were found located in the first three exons encoding a signal peptide and the highly conserved furin-like cysteine-rich domains. Study concluded and suggested that p.M1I variant is a recurrent mutation among Pakistani patients and can be considered as a polymorphism, having no effect on the observed disorder [87].

## 7. Conclusion and Perspectives

The functional complexity of Wnt signaling pathway has driven the interest of leading researchers towards in-depth analysis of Wnt signaling controlling factors. The extracellular antagonists and agonists provide defined regulation of signal and their further transmission. Herein, we have tried to summarize the various reported polymorphisms in known antagonists (sFRP, DKK, and Wif-1) and agonists (Rspo) of Wnt signaling pathway leading to the observed diseased state in humans (Table 1). As discussed in above sections both antagonists and agonists possess polymorphisms and have been linked to deleterious effects on human health. Therefore, the detailed genetic study of these antagonists and agonists may serve as a useful tool for understanding the development and progression of various diseases. Furthermore, genetic identification of Wnt signaling antagonists, responsible for disease susceptibility, remains at priority attention for cell biologists. Although extensive work has been carried out in recent years, nevertheless, we still need to understand the association of polymorphism in genes of antagonists and the progression of diseases. Advancements in this area may provide an insight and tend to open new opportunities for the early identification and treatments for yet incurable diseases.

## Figures and Tables

**Table 1:** SNPs in the selected genes and associated clinical phenotypes.

Gene	Chromosome	SNP ID	Geographical cohort	Phenotype	Reference
*SOST* *DC1 *	7p21.1	rs16878759 rs12699800 rs17619769	China	Osteoporosis	[23]

*SOST *	17q21.3	rs851054 rs851056	Australia	BMD and BMC at total hip	[18]

*SOST *	17q21.3	rs1513670 rs7220711 rs1107748	Iceland, Denmark, and Australia	Hip BMD associated	[30]

*SOST *	17q21.3	rs1230399	China	Spine, FN, trochanter, and total hip BMD	[31]

*sFRP3 *	2q32.1	rs288326 rs7775	Caucasian, Spanish	Knee osteoarthritis, hip osteoarthritis	[32–34]

*sFRP3 *	2q32.1	rs7775	Saudi	Breast cancer	[35]

*DKK1 *	10q21	rs1569198	Caucasian	Hip axis length	[36]

*DKK3 *	11p15.3	rs6485350	Saudi	Breast cancer	[35]

*DKK3 *	11p15.3	rs1472189	Japan	Renal cancer	[37]

*DKK4 *	8p11.1	rs3763511	Saudi	Breast cancer	[35]

## Abbreviations

LRP 5/6: Low density lipoprotein receptor-related protein 5/6
Fz: Frizzled receptor protein
GSK3 *β*: Glycogen synthase kinase 3 beta
APC: Adenomatosis polyposis coli protein
CKI*α*: Cyclin-dependent kinase inhibitor alpha
*β* TrCp: Beta-transducin repeat containing E3 ubiquitin protein ligase
WNT: Wingless-type MMTV integration site protein family
TCF/LEf1: T-cell factor/lymphoid-enhancing factor type 1
DVL: Dishevelled protein
Rho: Ras-like proteins
ROCK: Rho-associated kinase
ROR2: Receptor tyrosine kinase-like orphan receptor 2
JNKs: c-Jun N-terminal kinases
DKK: Dickkopf
sFRP: Secreted frizzled-related protein
Kremen: Kringle containing transmembrane protein
Wif: Wnt inhibitory factor
Rspo: R-spondin
LGR: Leucine-rich repeat-containing, G protein-coupled receptor
OMIM: Online mendelian inheritance in man.
